# Conundrums in neurology: diagnosing serotonin syndrome – a meta-analysis of cases

**DOI:** 10.1186/s12883-016-0616-1

**Published:** 2016-07-12

**Authors:** Ursula Werneke, Fariba Jamshidi, David M. Taylor, Michael Ott

**Affiliations:** Sunderby Research Unit – Psychiatry, Department of Clinical Sciences, Umeå University, Umeå, Sweden; Sunderby Hospital, 97180 Luleå, Sweden; Maudsley Hospital, Pharmacy Department Denmark Hill, King’s College London Institute of Pharmaceutical Science, London, UK; Department of Public Health and Clinical Medicine – Medicine, Umeå University, Umeå, Sweden

**Keywords:** Serotonin syndrome, Serotonin toxicity, Antidepressive agents, Drug interactions, Diagnosis, Differential, Neuroleptic malignant syndrome, Criteria, Meta-analysis

## Abstract

**Background:**

Serotonin syndrome is a toxic state, caused by serotonin (5HT) excess in the central nervous system. Serotonin syndrome’s main feature is neuro-muscular hyperexcitability, which in many cases is mild but in some cases can become life-threatening. The diagnosis of serotonin syndrome remains challenging since it can only be made on clinical grounds. Three diagnostic criteria systems, Sternbach, Radomski and Hunter classifications, are available. Here we test the validity of four assumptions that have become widely accepted: (1) The Hunter classification performs clinically better than the Sternbach and Radomski criteria; (2) in contrast to neuroleptic malignant syndrome, the onset of serotonin syndrome is usually rapid; (3) hyperthermia is a hallmark of severe serotonin syndrome; and (4) serotonin syndrome can readily be distinguished from neuroleptic malignant syndrome on clinical grounds and on the basis of medication history.

**Methods:**

Systematic review and meta-analysis of all cases of serotonin syndrome and toxicity published between 2004 and 2014, using PubMed and Web of Science.

**Results:**

Two of the four assumptions (1 and 2) are based on only one published study each and have not been independently validated. There is little agreement between current criteria systems for the diagnosis of serotonin syndrome. Although frequently thought to be the gold standard for the diagnosis of the serotonin syndrome, the Hunter criteria did not perform better than the Sternbach and Radomski criteria. Not all cases seem to be of rapid onset and only relatively few cases may present with hyperthermia. The 0 differential diagnosis between serotonin syndrome and neuroleptic malignant syndrome is not always clear-cut.

**Conclusions:**

Our findings challenge four commonly made assumptions about serotonin syndrome. We propose our meta-analysis of cases (MAC) method as a new way to systematically pool and interpret anecdotal but important clinical information concerning uncommon or emergent phenomena that cannot be captured in any other way but through case reports.

**Electronic supplementary material:**

The online version of this article (doi:10.1186/s12883-016-0616-1) contains supplementary material, which is available to authorized users.

## Background

Serotonin syndrome (SS) is a toxic state caused by serotonin (5HT) excess in the central nervous system (CNS). SS’s main feature is neuro-muscular hyperexcitability, which, if severe, can become life-threatening. The syndrome is thought to arise from 5HT_1A_ and 5HT_2_ receptor stimulation and has been linked to variety of drugs with direct or indirect serotonergic actions c1]. The risk of SS is higher when two or more serotonergic drugs are used in conjunction but cases caused by a single serotonergic agent have also been reported [1, 2]. The list of drugs associated with serotonergic toxicity is long, although experts do not always agree. Examples include antidepressants, lithium, opiates such as tramadol and meperidine (pethidine), dextromethorphan, some antiemetics such as metoclopramide and 5HT_3_ receptor antagonists (“setrons”). Non-antidepressant agent with monoamine oxidase (MAO) inhibiting properties such as MAO-B inhibitors for the treatment of Parkinson’s disease, the antibiotic linezolid or the contrast dye and methylene blue can also provoke serotonin excess and SS. Herbal medicines such as St John’s wort (hypericum perforatum) and illicit substances such as lysergic acid diethylamide (LSD) and 3,4-methylendioxy-methamphetamine (MDMA) are further examples. Even migraine medicines such as triptans have been implicated, though opinions remain divided [3, 4].

The diagnosis of SS remains challenging since it can only be made on clinical grounds. There is no objective diagnostic test. Three diagnostic classification systems are available, the Sternbach (SC), Radomski (RC) and Hunter (HC) criteria. All three classification systems try to reflect symptoms and symptom constellations thought to be indicative of SS. Whereas SC and RC draw on neuromuscular, cognitive and autonomous symptoms, HC focuses on neuromuscular symptoms such as clonus in its various forms, hyperreflexia and tremor [5–7] (Table 1).

**Table 1:** Sternbach, Radomski and Hunter diagnostic criteria

Sternbach	Radomski		Hunter
Co-incidence with the addition or increase in a known serotonergic agent to an established treatment regimen, at least three of the following features present:	Coincidence with the addition or increase in a known serotonergic agent (to an established treatment regimen), and the development of at least four minor or three major plus two minor symptoms:		In the presence of a serotonergic agent, symptom or symptom constellation:
Mental status changes (confusion, hypermania) Agitation Myoclonus Hyperreflexia Diaphoresis Shivering Tremor Diarrhea Incoordination Fever	Major	Minor	
	Mental		
	• Consciousness	• Restlessness	
	impairment	• Insomnia	• Spontaneous clonus • Inducible clonus AND agitation OR diaphoresis • Ocular clonus AND agitation OR diaphoresis • Tremor AND hyperreflexia • Hypertonic AND temperature > 38 °C AND ocular clonus OR inducible clonus
	• Elevated mood		
	• Semicoma/coma		
	Neurological		
	• Myoclonus	• Uncoordination	
	• Tremor	• Dilated pupils	
	• Shivering	• Akathisia	
	• Rigidity		
	• Hyperreflexia		
	Vegetative		
	• Fever	• Tachycardia	
	• Sweating	• Tachy/dyspnea	
		• Diarrhea	
		• Hyper/hypotension	
	• Clinical features not an integral part of the underlying psychiatric disorder prior to commencing the serotonergic agent.		
• Other aetiologies (e.g. infectious, metabolic or endocrine, substance abuse or withdrawal) have been ruled out.	• Other aetiologies (e.g. infectious, metabolic or endocrine, substance abuse or withdrawal) have been ruled out.		
• A neuroleptic drug had not been started or increased in dosage prior to the onset of the signs and symptoms listed above.	• A neuroleptic drug had not been started or increased in dosage prior to the onset of the signs and symptoms listed above.		

As SS is a relatively uncommon drug reaction, it cannot be picked up easily in randomized controlled trials (RCTs). Incidence estimates rely on adverse events reporting. Hence, the true incidence of SS is not known. Physicians may not even know about this condition. One survey among general practitioners (GPs) suggested an incidence of about 0.5 –1 cases per 1000 patient months of treatment. But this figure may have been an underestimate, since 85 % of the participating GPs were not familiar with SS [8]. We do not know how many cases of SS are mild, moderate or severe. Most cases of SS seem mild and self-limiting [8, 9]. In any event, SS more likely presents on a continuum rather than in clear-cut clinical stages [10]. Yet, failure to diagnose signs of serotonergic toxicity can turn mild and relatively harmless drug interactions into life-threatening catastrophic events.

Currently, we do not know how well the diagnostic classification systems agree with each other. Neither do we know which system performs best, despite claims that HC is superior [1, 11].

Here, we test four commonly held hypotheses regarding about the clinical features and aetiology of SS [1, 11], which have become established “textbook knowledge” despite their limited or partially biased evidence base.

- Hypothesis 1: HC performs clinically better than SC and RC.
- Hypothesis 2: In contrast to neuroleptic malignant syndrome (NMS), the onset of SS syndrome is usually rapid.
- Hypothesis 3: Hyperthermia is a hallmark of severe SS.
- Hypothesis 4: SS can readily be distinguished from NMS on clinical grounds and on the basis of medication history.

## Methods

We conducted a synopsis and a meta-analysis of all cases published between 2004 and 2014. As far as possible, we have adhered to the PRISMA guidelines in our method (Additional file 1).

### Search strategy

We searched PubMed and Thomson Reuter’s Web of Science for all cases of likely SS, using two keywords “serotonin syndrome” *or* “serotonin toxicity” and included all cases published between 1st January 2004 and 31st December 2014. We chose the year 2004 as a cut-off point, because by that time *all* three classification systems were available to clinicians.

### Eligibility criteria and case selection

We included all cases of adult patients meeting the definition of at least one of the three diagnostic systems and in which after differential diagnostic consideration SS emerged as the most likely diagnosis. We excluded all cases (1) not meeting *any* of the diagnostic criteria despite claiming a diagnosis of SS; (2) being etiologically uncertain despite meeting the diagnostic criteria; (3) containing insufficient clinical information to rate; (4) being historical; or (5) implicating first-generation antipsychotics or concomitant Neuroleptic Malignant Syndrome (NMS) (Additional file 2).

We abstracted all eligible cases into a new dataset, including general patient characteristics, onset, clinical course, mode of presentation, symptoms, diagnostic criteria, associated medications, treatment and outcome. Two investigators (UW and FJ or UW and MO) independently double-rated all cases regarding HC, SC and RC.

### Data item definitions and statistical analysis

#### Hypothesis 1

We established and compared the frequency of the 20 symptoms, appearing in *any* of the three diagnostic criteria sets. As proxies for severity of SS, we used rhabdomyolysis, defined by a creatine kinase ≥ 1500 mU/L (25.5 μkat/L) *or* intensive care treatment.

We calculated the overall agreement between the different diagnostic systems and estimated agreement beyond chance with Cohen’s kappa [12]. Then, we determined how many severe cases would have been missed by each criteria set. We used one-way ANOVA to determine whether there was a linear trend regarding reporting cases according HC, SC or RC over time (between 2004 and 2014).

#### Hypothesis 2

We defined “time to onset” as the time between the purported causative action and emergence of first symptoms of SS. We compared time to onset of “acute or invasive” cases with “sub-acute or non-invasive cases”. The acute or invasive category included surgery/trauma cases, overdoses and substance abuse. The sub-acute and non-invasive category included internal medicine and psychiatry cases.

#### Hypothesis 3

We included all cases with information on body temperature and established in how many cases fever or hyperthermia was present. We defined fever as a temperature > 38 °C (100.4 °F) (3) and hyperthermia as a temperature > 41.1 °C (106.0 °F) (5). We also included cases that explicitly stated “fever”, but did not give a temperature reading.

#### Hypothesis 4

We explored the frequencies of symptoms, which could suggest either, SS or NMS. We then looked at the top ten medications or drug combinations associated with SS.

## Results

In the final data set we included 299 cases (Additional file 3). 15.4 % cases related to intentional overdoses. 14 % of cases had resulted in rhabdomyolysis and 6.4 % in death. Overall, 39.2 % of 291 patients, for whom information on treatment was available, required intensive care (ICU).

### Hypothesis 1: HC performs clinically better than SC and RC

Confusion/consciousness impairment and agitation predominated as mental status changes. Of neurological symptoms, tremor and hyperreflexia were most frequently reported followed by muscle rigidity/hypertonia. Myoclonus was more common than clonus. Tachycardia, hypertension and fever were the most common autonomic symptoms. In cases with rhabdomyolysis, muscle rigidity/hypertonicity, fever and hyperthermia were significantly more frequent. In cases requiring intensive care, clonus, rigidity/hypertonicity, elevated temperature, fever, hyperthermia and tachy/dyspnea significantly presented more often (Table 2).

**Table 2:** Prevalence of symptoms of serotonin syndrome

	All (*n* = 299)	Rhabdo-myolysis (*n* = 42)	No rhabdo-myolysis (*n* = 257)	Intensive care (*n* = 114)	No inten-sive care (*n* = 177)
% Mental status changes					
Mania	2.7	2.4	2.7	-	4.5
Confusion/consciousness impairment/semi coma	63.9	73.8	62.3	68.4	59.9
Coma	11.4	21.4	9.7	25.4^**^	2.8
Agitation/restlessness	56.1	45.2	58.0	60.5	53.7
Insomnia	9.7	-^*^	11.3	2.6^**^	14.7
% Neurological symptoms					
Clonus • Spontaneous • Inducible	34.1 27.1 7.0	31.0 26.2 4.8	34.6 27.2 7.4	45.6^**^ 38.6^**^ 7.0	27.7 20.9 6.7
Eye clonus/roving eye movements • Spontaneous • Inducible	7.7 7.4 0.3	11.9 11.9 -	7.0 6.6 0.4	10.5 10.5 -	5.7 5.1 0.6
Myoclonus	41.5	35.7	42.4	43.9	39.5
Tremor	58.5	57.1	58.9	42.1^**^	70.1
Hyperreflexia	56.5	66.7	54.9	60.5	54.2
Rigidity/hypertonicity	45.4	59.5^*^	43.2	55.3^*^	40.6
Incoordination	15.1	4.8^*^	16.7	8.8^*^	19.8
% Vegetative symptoms					
Diarrhea	15.1	7.1	16.7	13.2	17.5
Fever^a^	59.7	76.9^*^	56.0	74.0^**^	46.6
Hyperthermia > 41.1 °C^b^	9.2	20.6^*^	6.5	17.6^*^	1.2
Diaphoresis	53.2	52.4	53.3	50.9	54.8
Shiver	15.1	16.7	14.8	19.3	12.4
Mydriasis	34.1	42.9	32.7	36.0	32.8
Tachy/bradycardia^c^	85.1	88.9	84.3	86.3	84.2
Hyper/hypotension^d^	75.8	76.6	75.7	80.7	70.6
Tachy/dyspnea as measured by a breathing rate > 20 or hypoxia	26.4	38.1	24.5	43.9^**^	15.9

^*^significant at *p* ≤ 0.05, ^**^significant at *p* ≤ 0.01

^a^221 of all patients, 39 cases with rhabdomyolysis and 182 without, 100 cases with intensive care and 116 without, for whom temperature was explicitly mentioned

^b^173 of all patients, 34 cases with rhabdomyolysis and 139 without, 85 cases with intensive care and 84 without, for whom actual temperature values were recorded

^c^221 of all patients, 36 cases with rhabdomyolysis and 185 without, 95 cases with intensive care and 120 without, for whom actual pulse values were recorded

^d^194 of all patients, 30 cases with rhabdomyolysis, 164 without, 88 cases with intensive care and 102 without, for whom actual blood pressure values were recorded

When we applied all three classification systems to all cases in our collection, we found that of the 299 cases, 48.8 % met all three diagnostic systems, 27.8 % both SC and RC, 13.7 % SC only, 5.4 % HC only, 2.3 % both HC and SC, 1.0 % both HC and RC and 1.0 % RC only. Reporting according to diagnostic criteria changed over time for HC and SC with a significant linear trend towards HC (*p* = 0.02) and away from SC (*p* ≤ 0.05). Reporting according to RC remained stable over time.

Agreement beyond chance between HC and SC and HC and RC, as measured by Cohen’s kappa, was poor for the whole sample and the subsets of cases with rhabdomyolysis and intensive care. The agreement beyond chance between SC and RC was fair for the whole sample and cases with rhabdomyolysis. It was moderate for intensive care cases (Table 3).

**Table 3:** Agreement between the three classification systems

Cases	n	Observed agreement (%)			Agreement beyond chance (Cohen’s k)		
		HC vs. SC	HC vs. RC	SC vs. RC	HC vs. SC	HC vs. RC	SC vs. RC
All	299	51.8	63.2	81.9	−0,10	0.20	0.30
Intensive care	114	61.4	61.4	91.2	−0.04	−0.01	0.45
Rhabdomyolysis	42	64.3	69.1	90.5	0.04	0.20	0.29

HC identified fewer overdoses, rhabdomyolysis and intensive care cases than SC or RC. In total, 35.7 % of all rhabdomyolysis and 35.1 % of all intensive care cases would not have been diagnosed as SS, adhering strictly to HC. In the subset of cases published between 2010 and 2014, 22.7 % of rhabdomyolysis and 26.4 % of intensive care cases would have been missed, adhering strictly to HC.

### Hypothesis 2: In contrast to neuroleptic malignant syndrome (NMS), the onset of SS syndrome is usually rapid

In our review of 236 (78.9 %) cases, for whom information on time to onset of SS was available, only 27.5 % of cases presented within 6 h and 44.5 % after 24 h. In total, 40.1 % belonged to the “acute/invasive treatment” group where pro-serotonergic drugs were administered quickly and/or in large doses (including overdoses). In this group, 52.2 % of cases presented within 6 h and 19.6 % after 24 h. 59.9 % belonged to the “sub-acute/non-invasive” group where medications were more gradually titrated and cross–tapered. In this group, only 11.8 % of cases presented within 6 h and 60.4 % after 24 h. All group differences were statistically significant with *p* ≤ 0.01.

### Hypothesis 3: Hyperthermia is a hallmark of severe SS

In our sample, where temperature was explicitly mentioned, fever had occurred 59.7 %. Of all cases with explicit temperature readings, only 9.2 % had temperatures consistent with hyperthermia. Fever and hyperthermia were significantly more common in cases with rhabdomyolysis or those admitted to intensive care. Nonetheless, about one quarter of intensive care cases would have been missed relying on fever alone. About four fifths would have been missed relying on hyperthermia alone (Table 2).

### Hypothesis 4: SS can readily be distinguished from neuroleptic malignant syndrome (NMS) on clinical grounds and on the basis of medication history

In our case collection, ten causes accounted for 83.3 % of all SS cases. Combination of antidepressants with opiates and overdoses were most common. These ten causes accounted for 86.8 % of SS cases requiring intensive care. Here, overdoses and combinations of antidepressants with methylene blue, opiates or linezolid emerged as the most frequent causes (Table 4). Ten cases implicated second-generation antipsychotics (SGA) as the decisive triggering factor. Eight of these had occurred in combination with antidepressants and two in the context of SGA swap.

**Table 4:** Usual suspects? Top 10 causes of serotonin syndrome

Cause	All cases (*n* = 299)	Accounting for ICU cases (*n* = 114)
	%	%
Combination antidepressant - opiate	16.1	10.5
Overdose	15.4	26.3
Combination antidepressant with another potentially serotonergic agent^a^	13.0	7.9
Combination of different antidepressants	7.7	5.3
Combination antidepressant - linezolid	7.0	9.6
Swap antidepressants	6.4	4.4
Combination antidepressant – methylene blue	5.4	11.4
Start of antidepressants	4.7	-
Substance misuse	4.3	8.8
Start/ discontinuation of second generation antipsychotics in the presence of another pro-serotonergic agent^a^	3.3	2.6

^a^Including agents such as dextromethorphan, SNRI used for other purposes (milnacipran, sibutramine), triptanes, antiemetics (metoclopramide, setrones); other agents affecting the metabolism of antidepressants

Rigidity and rhabdomyolysis, symptoms commonly associated with NMS, were also frequently seen in our sample of SS. Rigidity/hypertonicity had occurred in 45.4 % and rhabdomyolysis in 14.0 % (Table 2).

## Discussion

Our findings challenge four commonly made assumptions about SS, which over time have become accepted textbook knowledge. There is only little agreement between current criteria systems for the diagnosis of SS so that clinicians need to keep an open mind about the diagnosis, even if diagnostic criteria not are met.

### Hypothesis 1

HC may clinically be less sensitive than hitherto assumed. We acknowledge that our case note collection is inevitably subject to selection bias, but this does not invalidate our arguments. Understanding uncommon or emergent conditions that cannot be captured through randomized controlled trials often depends on pattern recognition from few cases. This way, for instance, the acquired immune deficiency syndrome (AIDS) was discovered [13]. It is unlikely that *all* cases missed by HC were false positives. The HC originators themselves have reported 96 % specificity for SC (compared to 97 % for HC). This means we would only have expected 4 % false positive cases when applying SC. Neither is the trend towards reporting according to HC over time automatically a proof a HC superiority. This trend may simply reflect that it has become increasingly difficult to publish cases that do not fulfil HC.

#### History of the three classification systems

SC was the first classification system published in 1999, derived from 38 psychiatric inpatients [5]. SC was criticized for being too unspecific and relying too much on mental status changes. For instance, SC could indicate SS without any neuromuscular symptom, if a patient presented with confusion, agitation and elevated temperature.

In 2001, Radomski refined SC based on a review of 62 cases, including Sternbach’s original 38 cases [6]. RC differentiated between major and minor symptoms of SS and added rigidity to the neuromuscular symptoms.

In 2003, Dunkley et al. released the HC based on a review of 2222 cases of overdoses with selective serotonin reuptake inhibitors (SSRIs) [7]. This classification focused on neuromuscular symptoms to a far greater extent than the other two classification systems. HC introduced clonus in its various forms (spontaneous, inducible and ocular). At the same time, HC removed myoclonus from the symptom list. The HC originators reported their classification system as more sensitive and specific than the other two classification systems.

#### Is there a gold standard for diagnosing SS?

Rather than being a tangible physical quantity such as body temperature or blood glucose, SS is an abstract construct made up of various conceptual, elements (items). In this way, the three classification systems are similar to a psychometric scale that might measure a construct such as quality of life. As any psychometric measurement tool, all three classification systems have tried in various ways to identify symptoms or symptom constellations that capture best “the nature of what is being measured” and “the relationship of that variable to its purported cause” [14]. In the case of SS, we measure CNS hyperexcitability and try to relate this to a purported drug-induced serotonin excess.

As CNS hyperexcitability can manifest itself in many ways, it may be difficult to establish a “true” gold standard for the diagnosis of SS. HC, the latest classification system in use, has reported superior sensitivity and specificity though. In these terms, HC should be best at *both* picking up cases and not picking up false positive cases. This has led to wide-spread endorsement of HC as the gold standard for the diagnosis of SS [1, 11]. It has also been suggested that reports of cases of SS that have not met HC are of poor scientific value [15]. Yet, the purported HC superiority is based on *one* study only. This may not be sufficient to underpin HC superior validity since “the burden of evidence in testing construct validity arises not from a single powerful experiment, but from a series of converging experiments” [14].

One concern regarding validity is that HC was derived exclusively from SSRI overdoses. Although HC is based on many more cases than SC and RC, the confinement to overdose cases suggests that HC may not automatically be generalizable to non-overdose states of serotonin excess, where symptoms may be less clear-cut than in acute poisonings. A second concern is that a proportion of the cases used to derive HC was then also used to validate HC. Verifying a construct, in this case SS, by including the same data, which was used to derive the construct in the first place, will lead to an overestimate of its validity [16]. Thirdly, HC may not perform well in patients with other underlying neurological pathologies. Hyperreflexia or clonus, two essential HC symptoms, may not occur in patients with peripheral neuropathy where nerve damage “masks upper motor neuron signs” [17]. Equally, reflexes or clonus may not be elicitable in patients with severe SS who have developed substantial muscle rigidity [1].

Clinically, particularly when a condition is life threatening, it may be better to err on the side of caution and temporarily withdraw a purported offending agent, until the differential diagnosis is clarified and appropriate action can be taken. The alternative of refusing to take into account symptoms because they do not meet HC and continuing a potentially harmful agent seems less safe. In many such cases, it may be possible to reinstate treatment with serotonergic drugs, once the SS has resolved and measures are taken to prevent the precipitating event in the future. Such measures include avoiding future overdoses, discontinuing opiates with serotonergic properties or withdrawing serotonergic antidepressants in good time prior to administration of methylene blue and linezolid.

### Hypothesis 2

The claim that contrary to neuroleptic malignant syndrome (NMS), the onset of SS is usually rapid is based on *one* review of 41 cases with SS published between 1995 and 1999 [18]. In this case collection, 61.5 % presented with six hours of ingestion of the causative agent and only 25.6 % later than 24 h. Thus, SS may develop quickly *or* slowly, depending on the context in which it occurs. Our results fit with the observation that the onset of linezolid-associated SS may be delayed in elderly [19]. Whether SS develops quickly or slowly, may to a large extent depend on pharmacokinetic factors. Although polypharmacy is an important etiological factor in the development of SS *per se*, dose and speed of distribution may determine its severity. It remains also uncertain whether SS typically resolves much faster than NMS [20]. Rather, time to SS resolution may depend on the half-lives of the implicated agents.

### Hypothesis 3

Fever is considered a hallmark of SS and hyperthermia. To be more precise, a temperature > 41.1 °C, a hallmark of *severe* SS [1]. Elevated temperature in SS is thought to arise from a loss of physiologic control of temperature regulation (leading to hyperthermia) rather than pyrogen mediated upregulation of the hypothalamic thermostat (pyrexia/hyperpyrexia) [20]. It is unclear, why some SS cases develop hyperthermia and others do not. In the context of SS, hyperthermia is linked to increased muscle activity as consequence of hyperexcitability and direct serotonergic effects on the muscle [1, 21, 22]. Naturally, these factors are subject to biological variability. Psychological and environmental factors such as exercise, heating, apprehension and excitement have shown to precipitate serotonin-mediated hyperthermia in susceptible animals [22].

### Hypothesis 4

We tend to think of neuroleptic malignant syndrome in the context of antipsychotics and of serotonin syndrome in the context of antidepressants. Yet, the distinction between NMS and SS is less clear-cut in agents which have *both*, antidopaminergic and serotonergic, properties. Possibly, NMS and SS are part of the same pathology rather than two different pathological entities. This could explain why rigidity/hypertonicity has emerged as a key symptom of SS. This could also explain why even severe SS is associated with rhabdomyolyis. SS and NMS are both associated with neuromuscular hyperexcitability. Subcortical dopaminergic and serotonergic nuclei anatomically overlap and share many afferent and efferent projections [23]. Indeed, the “atypicality” of SGAs is to some extent based on their 5HT_2_ antagonistic and 5HT_1_ agonistic properties, which may allow more dopaminergic activity in the nigrostriatal system [24]. Sudden changes in SGA mediated serotonergic activity may precipitate extreme changes in dopamine neurotransmission, resulting in symptoms usually associated with NMS.

## Conclusions

Our findings challenge four commonly made assumptions about SS, which over time have become accepted knowledge. Two of these assumptions were based each on one published study which was then successively quoted. As HC may not be superior to the other available classification systems clinicians should keep an open mind about the diagnosis, even if HC are not met. Not all cases are of rapid onset and only relatively few cases present with hyperthermia. The differential diagnosis between SS and NMS is not always clear-cut. Both conditions overlap in symptoms and many drugs have both dopamine and serotonin modulating properties.

For the diagnosis of SS, focussing on aetiology may be more important than relying on symptoms or symptom constellations. Aetiological considerations are important, because patients with symptoms of serotonin syndrome turn to emergency rooms for help rather than the original prescriber. Understanding the aetiology is also crucial when deciding on whether to re-challenge with a purported offending agent or not.

On the one hand, it is important to withhold drugs likely to cause serious adverse effects. On the other hand, it is important not unnecessarily to withhold medicines patients need for exaggerated fear of serotonin syndrome.

Whereas methods for reviewing intervention and epidemiological studies are well developed, there are no comparable accepted methods for reviewing anecdotal clinical data. Ignoring such data due to methodological concerns such as selection bias is not an option, when uncommon but clinically significant phenomena can only be identified from cases. Indeed, up to 50 % of our current guidelines may largely rely on lower level evidence and expert opinions [25, 26]. We propose our method to meta-analyse cases (MAC) as a new way systematically to collate and analyse anecdotal, but important, clinical information. This way, we may be able to improve the story we can tell from the evidence at hand [27].

## Abbreviations

5HT, 5-hydroxytryptamine; AIDS, acquired immune deficiency syndrome; ANOVA, analysis of variance; C, Celsius; CNS, central nervous system; Cohen’s K, Cohen’s kappa; F, Fahrenheit; GP, general practitioner; HC, Hunter criteria; ICU, intensive care unit; LSD, lysergic acid diethylamide; MAC, meta-analysis of cases; MAO, monoamine oxidase; MDMA, 3,4-methylendioxy-metamphetamine; NMS, neuroleptic malignant syndrome; PRISMA, Preferred reporting items for systematic reviews and meta-analyses; RC, Radomski criteria; RCT, randomized controlled trial; SC, Sternbach criteria; SGA, second generation antipsychotic; SS, serotonin syndrome; SSRI, selective serotonin reuptake inhibitor

## Additional files

Additional file 1: Method for meta-analysis of our cases (MAC) adapted to the PRISMA checklist, table. (DOCX 118 kb) Additional file 2: Flow diagramme – identification of cases with serotonin syndrome, flow diagramme. (DOCX 99 kb) Additional file 3: List of 299 cases derived from 257 articles, reference list for data set. (DOCX 98 kb)
