# Supplementary material for: Conundrums in neurology: diagnosing serotonin syndrome – a meta-analysis of cases
Source: BMC Neurol. 2016 Jul 12;16:97. doi: 10.1186/s12883-016-0616-1 (PMC4941011; doi:10.1186/s12883-016-0616-1)
Supplement: Additional file 1: — Method for meta-analysis of our cases (MAC) adapted to the PRISMA checklist, table. (DOCX 118 kb) [file 12883_2016_616_MOESM1_ESM.docx]

**Appendix 1**

**Method for meta-analysis of our cases (MAC) adapted to the PRISMA checklist***

| **PRISMA requirement** | **#** | **Our review** |
| --- | --- | --- |
| Title and abstract | | |
| Title: identify the report as a systematic review, meta-analysis, or both | 1 | Conundrums in neurology: diagnosing serotonin syndrome – a meta-analysis of cases |
| Abstract | | |
| Structured summary | 2 | In the framework of a BMC Neurology debate article |
| Introduction |  |  |
| Rationale | 3 | Given |
| Objective | 4 | Explicit outline of four hypothesis to be tested |
| Methods | | |
| Protocol and registration | 5 | Not applicable |
| Eligibility criteria | 6 | We screened for case reports and case series reporting likely serotonin syndrome (SS). We included all cases of adult patients meeting the definition of at least one of the three diagnostic systems and in which after differential diagnostic consideration SS emerged as the most likely diagnosis. We excluded all cases (1) not meeting *any* of the diagnostic criteria despite claiming a diagnosis of SS; (2) being etiologically uncertain despite meeting the diagnostic criteria; (3) containing insufficient clinical information to rate; (4) being historical; or (5) implicating first-generation antipsychotics or concomitant neuroleptic malignant syndrome (NMS). |
| Information sources | 7 | PubMed and Thomson Reuter’s Web of Science for all cases of likely SS |
| Search | 8 | Search terms “serotonin syndrome” or “serotonin toxicity”  Limits: Since 2004, by which time Hunter criteria had been published and all three diagnostic criteria were available. |
| Study selection | 9 | Inclusion and exclusion criteria as above |
| Data collection process | 10 | We abstracted all eligible cases into a new dataset, including general patient characteristics, onset, clinical course, mode of presentation, symptoms, diagnostic criteria, associated medications, treatment and outcome. Two investigators (UW and FJ or UW and MO) independently double-rated all cases regarding Hunter (HC), Sternbach (SC) or Radomski (RC) criteria. |
| Data items | 11 | Symptoms of SS: 20 symptoms appearing in *any* of the three diagnostic criteria sets.  Fever defined as temperature > 38°C (100.4°F) Hyperthermia defined as temperature > 41.1°C (106.0°F).  Time to onset: time between the purported causative action and emergence of first symptoms of SS.  Severe SS: Either rhabdomyolysis, defined by a creatine kinase ≥ 1500 mU/L (25.5 µkat/L) or intensive care treatment. |
| Bias | 12 | Potential for bias comparable to other works in this area, e.g. HC are derived from and tested only on SSRI overdoses and not on non-overdose cases, independent validation of HC is not available and part of the of the cases used to derive HC was also used to validate HC.  *Source of bias in our study*  At publication level: publication bias favoring unexpected or uncommon cases.  *Attempts to minimize bias in our study*  At study level: two investigators (UW and FJ or UW and MO) independently double-rated all cases regarding HC, SC and RC.  At outcome level: test for linear trend regarding reporting cases according HC, SC or RC over time (between 2004 and 2014). |
| Summary measures | 13 | Agreement beyond chance measured by Cohen’s kappa  Z tests for independent and dependent groups to compare proportions.  One-way ANOVA to determine whether there was a linear trend regarding reporting cases according HC, SC or RC over time (between 2004 and 2014). |
| Synthesis of results | 14 | Cf. #13. |
| Risk of bias across studies | 15 | Discussed in the context of developing methods for the review of anecdotal data to describe uncommon conditions, which do not yield themselves to large observational studies or RCTs |
| Additional analysis | 16 | Subgroup analysis for cases with severe SS, cases due to overdose and cases resulting in death. |
| Results | | |
| Study selection | 17 | Cf. flow diagramme in appendix 2b |
| Study characteristics | 18 | Individual case records or case series |
| Risk of bias within studies | 19 | Double rating of all cases |
| Results of individual studies | 20 | Not applicable to individual cases |
| Synthesis of results | 21 | Statistical tests of all four hypothesis |
| Risk of bias across studies | 22 | Cf. #12 |
| Additional analysis | 23 | Cf. #16 |
| Discussion | | |
| Summary of evidence | 24 | Discussion in the main text |
| Limitations | 25 | Discussion in the main text |
| Conclusions | 26 | Provided in the main text |
| Funding |  |  |
| Funding | 27 | This work was supported by a grant of the Norrbotten County Research & Development Fund, Sweden. |

*Moher D, Liberati A, Tetzlaff J, Altman DG; PRISMA Group. Preferred reporting items for systematic reviews and meta-analyses: the PRISMA statement. BMJ. 2009 Jul 21;339:b2535. doi: 10.1136/bmj.b2535.
