# Supplementary material for: Conundrums in neurology: diagnosing serotonin syndrome – a meta-analysis of cases
Source: BMC Neurol. 2016 Jul 12;16:97. doi: 10.1186/s12883-016-0616-1 (PMC4941011; doi:10.1186/s12883-016-0616-1)
Supplement: Additional file 2: — Flow diagramme – identification of cases with serotonin syndrome, flow diagramme. (DOCX 99 kb) [file 12883_2016_616_MOESM2_ESM.docx]

**Appendix 2: Flow diagramme – Identification of cases with serotonin syndrome**

Thomson Reuters’ Web of Science

“Serotonin syndrome” or “serotonin toxicity”

n = 799

Pub Med

“Serotonin syndrome” or “serotonin toxicity”

n = 689

Potentially eligible cases from 323 articles:

n = 394

Excluded cases from 70 articles

n = 95

Not enough information: 19

Did not meet *any* criteria set: 55

Diagnosis unclear/ not made: 9

Neuroleptics/first generation antipsychotics or comorbid NML: 9

Historical cases: 3

Cases included from 257 articles

n = 299
