# Supplementary material for: Conundrums in neurology: diagnosing serotonin syndrome – a meta-analysis of cases
Source: BMC Neurol. 2016 Jul 12;16:97. doi: 10.1186/s12883-016-0616-1 (PMC4941011; doi:10.1186/s12883-016-0616-1)
Supplement: Additional file 3: — List of 299 cases derived from 257 articles, reference list for data set. (DOCX 98 kb) [file 12883_2016_616_MOESM3_ESM.docx]

**Appendix 3:**

**List of 299 cases derived from 257 articles**

1. Adan-Manes J, Novalbos J, Lopez-Rodriguez R, Ayuso-Mateos JL, Abad-Santos F. Lithium and venlafaxine interaction: a case of serotonin syndrome. J Clin Pharm Ther. 2006;31:397-400.
2. Agell I. Serotonin syndrome resulting from switching antidepressants in a patient with chronic pain. A case report. Eur Psychiatry. 2008;23:S199S.
3. Ailawadhi S, Sung KW, Carlson LA, Baer MR. Serotonin syndrome caused by interaction between citalopram and fentanyl. J Clin Pharm Ther. 2007;32:199-202.
4. Alkhatib AA, Peterson KA, Tuteja AK. Serotonin syndrome as a complication of fentanyl sedation during esophagogastroduodenoscopy. Dig Dis Sci. 2010;55:215-6.
5. Alnwick GM. Misdiagnosis of serotonin syndrome as fibromyalgia and the role of physical therapists. Phys Ther. 2008;88:757-65.
6. Altman CS, Jahangiri MF. Serotonin syndrome in the perioperative period. Anesth Analg. 2010;110:526-8.
7. Altman EM, Manos GH. Serotonin syndrome associated with citalopram and meperidine. Psychosomatics. 2007;48(4):361-3.
8. Alvarez-Perez FJ, Roca M, Martorell E, Espino AM, Uson MM, Figuerola A et al. [Serotonin syndrome: report of two cases and review of the literature]. Rev Neurolog. 2005;40:159-62.
9. Ameen S, Praharaj SK. Functional auditory hallucinations in a case of serotonin syndrome. J NeuropsychClin Neurosci. 2013;25:E60-1.
10. Anonymous. Venlafaxine + tramadol: serotonin syndrome. Prescrire Int. 2004;13:57.
11. Anonymous. Fluoxetine + hydromorphone: serotonin syndrome? Prescrire Int. 2004;13:57.
12. Ansermot N, Hodel PF, Eap CB. Serotonin toxicity after addition of mirtazapine to escitalopram. J Clin Psychopharmacol. 2014;3:540-1.
13. Atasoy N, Ozturk D, Konuk N. Serotonin syndrome resulting from coadministration of venlafaxine, and mirtazapine. Eur Neuropsychopharmacol. 2008;18:S309-S10.
14. Attar-Herzberg D, Apel A, Gang N, Dvir D, Mayan H. The serotonin syndrome: initial misdiagnosis. Isr Med Assoc J. 2009; 11:367-70.
15. Bakim B, Sertcelik S, Tankaya O. A Case of Serotonin Syndrome with Antidepressant Treatment and Concomitant use of The Herbal Remedy (Peganum Harmala). Klinik Psikofarmakoloji Bulteni-Bull Clin Psychopharmacol. 2012;22:359-61.
16. Baptista G, Eiden C, Monguillot P, Philibert C, Jeandel C. Serotonin syndrome during treatment with low dose of escitalopram associated with miconazole mucoadhesive tablet: a suspected drug interaction. International psychogeriatrics / IPA. 2012;24:845-7.
17. Batista M, Dugernier T, Simon M, Haufroid V, Capron A, Fonseca S et al. The spectrum of acute heart failure after venlafaxine overdose. Clin Toxicol. 2013;51:92-5.
18. 25. Beatty NC, Nicholson WT, Langman LJ, Curry TB, Eisenach JH. Pharmacogenetic workup of perioperative serotonin syndrome. Journal of clinical anesthesia. 2013;25(8):662-5. doi:10.1016/j.jc1inane.2013.06.005.
19. 26. Bergeron L, Boule M, Perreault S. Serotonin toxicity associated with concomitant use of linezolid. The Annals of pharmacotherapy. 2005;39(5):956-61. doi:10.1345/aph.1E523.
20. 27. Bertoli RB, Tosi M, Vanini G, Caduff P, Cerny A. Serotonin syndrome induced by mirtazapine monotherapy. Drug Saf. 2004;27(12):920-.
21. 28. Bertolin-Guillen JM, Climent-Diaz B, Navarre-Gimeno A. Serotonin syndrome due to association of venlafaxine, maprotiline and reboxetine. European psychiatry : the journal of the Association of European Psychiatrists. 2004;19(7):456-7. doi:10.1016/j.eurpsy.2004.06.012.
22. Bogdanovic Z, Nalamati JR, Kilcullen JK, Dhuper S. Antidepressant-induced adverse reactions in a patient with hemorrhagic stroke. Ann Pharmacother. 2005;39:1755-7.
23. Bordelon S, Brett Lloyd R, Rosenthal LJ. Serotonin syndrome and stiff-person syndrome: diagnostic challenges in psychosomatic medicine. Psychosomatics. 2014;55:506-11.
24. 31. Bosak A, LoVecchio F, Levine M. Recurrent seizures and serotonin syndrome following "2C-I" ingestion. J Med Tox. 2013;9:196-8.
25. Bosak AR, Skolnik AB. Serotonin syndrome associated with metaxalone overdose. J mwed Tox. 2014;10:402-5.
26. Boulanger-Gobeil C, St-Onge M, Laliberte M, Auger PL. Seizures and hyponatremia related to ethcathinone and methylone poisoning. J Med Tox. 2012;8:59-61.
27. Bramness JG, Morland J, Sorlid HK, Rudberg N, Jacobsen D. Carisoprodol intoxications and serotonergic features. Clin Toxicol. 2005;43(1):39-45.
28. Brown TM. Nitroglycerin in the treatment of the serotonin syndrome. The Am J Emerg Med. 2004;22:510.
29. Brvar M, Stajer D, Kozelj G, Osredkar J, Mozina M, Bunc M. Urinary serotonin level is associated with serotonin syndrome after moclobemide, sertraline, and citalopram overdose. Clinical Toxicology. 2007;45:458-60.
30. Bryant SM, Kolodchak J. Serotonin syndrome resulting from an herbal detox cocktail. Am J Emerg Med. 2004;22:625-6.
31. Bush E, Miller C, Friedman I. A case of serotonin syndrome and mutism associated with methadone. J Palliat Med. 2006;9:1257-9.
32. Butler MC, Di Battista M, Warden M. Sertraline-induced serotonin syndrome followed by mirtazapine reaction. Prog Neuro-Psych Biol Psych. 2010;34:1128-9.
33. Canan F, Korkmaz U, Kocer E, Onder E, Yildirim S, Ataoglu A. Serotonin syndrome with paroxetine overdose: a case report. Prim Care Companion J Clin Psych. 2008;10:165-7.
34. Cassens S, Nickel EA, Quintel M, Neumann P. The serotinin syndrome. Fatal course of intoxication with citalopram and moclobemide. Anaesthesist. 2006;55:1189.
35. Cekmen N, Badalov P, Erdemli O. [Seronin syndrome and cardiac arrest caused by high-dose moclobemide (case report)]. Anesteziol Reanimatol. 2008:64-5.
36. Chander WP, Singh N, Mukhiya GK. Serotonin syndrome in maintenance haemodialysis patients following sertraline treatment for depression. J Indian Med Associ. 2011;109:36-7.
37. Chen H-Y, Wu M-H, Lynn J-J, Chen H-C, Liao S-C. Delayed presentation of serotonin syndrome after co-ingestion of serotoninergic agents and benzodiazepines. Clin Toxi. 2013;51:319-20.
38. Cheng P-L, Hung S-W, Lin L-W, Chong C-F, Lau C-I. Amantadine-induced serotonin syndrome in a patient with renal failure. Am J Emerg Med 2008;26:112.e5-6.
39. Chirwa I, Savage M, Sarwar A, Norris A. Serotonin syndrome secondary to fluoxetine precipitated by radiation induced cerebral vasculopathy. Clin Med. 2008;8:107-8.
40. Choong K, Ghiculescu RA. Iatrogenic neuropsychiatric syndromes. Aust Family Physician. 2008;37:627-9.
41. Chopra P, Ng C, Schweitzer I. Serotonin syndrome associated with fluoxetine and olanzapine. Word J Biol Psychiatry. 2004;5:114-5.
42. Choudhury M, Hote MP, Verma Y. Serotonin syndrome in a postoperative patient. J Anaesthesiology, Clin Pharmacol. 2011;27:233-5.
43. Choudhury M, Hote MP, Verma Y. Serotonin syndrome in a postoperative cardiac surgical patient--a case report. Middle East J Anesthesiol. 2011;21:111-4.
44. Clark DB, Andrus MR, Byrd DC. Drug interactions between linezolid and selective serotonin reuptake inhibitors: Case report involving sertraline and review of the literature. Pharmacotherapy. 2006;26:269-76.
45. Coster S, Visser MH, Touw DJ, Wirtz PW. Serotonin syndrome with sertraline and indomethacin. J Clin Psychopharmacol. 2010;30:468-70..
46. Dagtekin O, Marcus H, Muller C, Bottiger BW, Spohr F. Lipid therapy for serotonin syndrome after intoxication with venlafaxine, lamotrigine and diazepam. Minerva Anestesiol. 2011;77:93-5.
47. Dardis C, Omoregie E, Ly V. Fatal serotonin syndrome precipitated by oxcarbazepine in a patient using an selective serotonin reuptake inhibitor. Neurologist. 2012;18:204-5.
48. Das PK, Warkentin DI, Hewko R, Forrest DL. Serotonin syndrome after concomitant treatment with linezolid and meperidine. Clin Inf Dis. 2008;46:264-5.
49. Davies O, Batajoo-Shrestha B, Sosa-Popoteur J, Olibrice M. Full recovery after severe serotonin syndrome, severe rhabdomyolysis, multi-organ failure and disseminated intravascular coagulopathy from MDMA. Heart Lung. 2014;43:117-9.
50. Davis JJ, Buck NS, Swenson JD, Johnson KB, Greis PE. Serotonin syndrome manifesting as patient movement during total intravenous anesthesia with propofol and remifentanil. J Clin Anesth. 2013;25:52-4.
51. Day LT, Jeanmonod RK. Serotonin syndrome in a patient taking Lexapro and Flexeril: a case report. Am J Emerg Med. 2008;26:1069 e1-3.
52. DeBellis RJ, Schaefer OP, Liquori M, Volturo GA. Linezolid-associated serotonin syndrome after concomitant treatment with citalopram and mirtazepine in a critically ill bone marrow transplant recipient. J Intensive Care Med. 2005;20:351-3.
53. Decoutere L, De Winter S, Vander Weyden L, Spriet I, Schrooten M, Tournoy J et al. A venlafaxine and mirtazapine-induced serotonin syndrome confirmed by de- and re-challenge. Int Journal Clinical Pharm. 2012;34:686-8.
54. Dolz Aspas R, Juyol Rodrigo MC, Gracia Sanchez P. [Drugs and serotonin syndrome]. An Med Interna. 2008;25:373-4.
55. Duval F, Flabeau O, Razafimahefa J, Spampinato U, Tison F. Encephalophaty associated with rasagiline and sertraline in Parkinson's disease: possible serotonin syndrome. Mov Disord. 2013;28:1464.
56. Dvir Y, Smallwood P. Serotonin syndrome: a complex but easily avoidable condition. General Hospital Psychiatry. 2008;30:284-7.
57. El-Okdi NS, Lumbrezer D, Karanovic D, Ghose A, Assaly R. Serotonin syndrome after the use of tramadol and ziprasidone in a patient with a deep brain stimulator for Parkinson disease. Am J Ther. 2014;21:e97-9.
58. Elizondo Armendariz JJ, Pellejero Hernando E, Noceda Urarte MM, Gutierrez Valencia M. [Probable serotonin syndrome due to linezolid and meperidine interaction]. Farm Hosp. 2012;36:448-9. doi:10.1016/j.farma.2011.10.006.
59. Esquivel Lopez A. Serotonin syndrome induced by rasagiline. Mov Disord. 2013;28:S219-S.
60. Evans CE, Sebastian J. Serotonin syndrome. Emerg Med J. 2007;24:e20.
61. Evans RW. The FDA alert on serotonin syndrome with combined use of SSRIs or SNRIs and Triptans: an analysis of the 29 case reports. Med Gen Med. 2007;9:48.
62. Falls BA, Gurrera RJ. Serotonin syndrome in a patient on tramadol, bupropion, trazodone, and oxycodone. Psychosomatics. 2014;55:305-9.
63. Fernandes C, Reddy P, Kessel B. Rasagiline-induced serotonin syndrome. Mov disord. 2011;26:766-7.
64. Ferra AC, Bosch PV, Raurich JM. Serotonin syndrome due to interaction between linezolid, tryptophan, and metoclopramide. Med Intensiv. 2009;33:360-1.
65. Feychting K, Jonsson B, Sjoberg G. Successful Treatment of Serotonin Syndrome with Sublingual Olanzapine. Clin Toxicol. 2012;50:323.
66. Fil L, Sud P, Falkoff M. Herbal remedy or herbal problem? Clin Toxicol. 2014;52:740.
67. Frank C. Recognition and treatment of serotonin syndrome. Can Fam Physician. 2008;54:988-92.
68. Freeman WD, Chabolla DR. 36-Year-old woman with loss of consciousness, fever, and tachycardia. Mayo Clin Proc. 2005;80:667-70.
69. Freijo Guerrero J, Tardon Ruiz de Gauna L, Gomez JJ, Aguilera Celorrio L. Serotonin syndrome after administration of mirtazapine in a critical care unit. Rev Esp Anestesiol Reanim. 2009;56:515-6.
70. Fugate JE, White RD, Rabinstein AA. Serotonin syndrome after therapeutic hypothermia for cardiac arrest: a case series. Resuscitation. 2014;85:774-7.
71. Ganetsky M, Babu KM, Boyer EW. Serotonin syndrome in dextromethorphan ingestion responsive to propofol therapy. Pediatr Emergency Care. 2007;23:829-31.
72. Ganetsky M, Bird SB, Liang IE. Acute myocardial infarction associated with the serotonin syndrome. Ann Int Med. 2006;144:782-3.
73. Garrett G, Sweeney M. The serotonin syndrome as a result of mephedrone toxicity. BMJ Case Rep. 2010; doi:10.1136/bcr.04.2010.2925.
74. Garrett PM. Tramadol overdose and serotonin syndrome manifesting as acute right heart dysfunction. Anaesth Intensive Care. 2004;32:575-7.
75. Gelener P, Gorgulu U, Kutlu G, Ucler S, Inan LE. Serotonin syndrome due to duloxetine. Clin Neuropharmacol. 2011;34:127-8.
76. Gnanadesigan N, Espinoza RT, Smith R, Israel M, Reuben DB. Interaction of serotonergic antidepressants and opioid analgesics: Is serotonin syndrome going undetected? J Am Med Dir Assoc. 2005;6:265-9.
77. Go AC, Golightly LK, Barber GR, Barron MA. Linezolid interaction with serotonin reuptake inhibitors: report of two cases and incidence assessment. Drug Metab Drug Interact. 2010;25:41-7.
78. Gollapudy S, Kumar V, Dhamee MS. A case of serotonin syndrome precipitated by fentanyl and ondansetron in a patient receiving paroxetine, duloxetine, and bupropion. J Clin Anesth. 2012;24:251-2.
79. Gressier F, Ellul P, Dutech C, Ait Tayeb Ael K, Monfort J, Corruble E et al. Serotonin toxicity in a CYP2D6 poor metabolizer, initially diagnosed as a drug-resistant major depression. Am J Psych. 2014;171:890.
80. Grubb KJ, Kennedy JL, Bergin JD, Groves DS, Kern JA. The role of methylene blue in serotonin syndrome following cardiac transplantation: a case report and review of the literature. J Thorac Cardiovasc Surg. 2012;144:e113-6.
81. Guo SL, Wu TJ, Liu CC, Ng CC, Chien CC, Sun HL. Meperidine-induced serotonin syndrome in a susceptible patient. Br J Anaesth. 2009;103:369-70.
82. Gupta V, Karnik ND, Deshpande R, Patil MA. Linezolid-induced serotonin syndrome. BMJ Case Rep. 2013;doi:10.1136/bcr-2012-008199.
83. Hachem RY, Hicks K, Huen A, Raad I. Myelosuppression and serotonin syndrome associated with concurrent use of linezolid and selective serotonin reuptake inhibitors in bone marrow transplant recipients. Clin Inf Dis. 2003;37:e8-11.
84. Hackelsberger N, Ried M, Reiners A. Drug Interaction - a Pitfall in Rehabilitation. Phys Med Rehab Kuror. 2008;18:355-7.
85. Hadikusumo B, Ng B. Serotonin syndrome induced by duloxetine. The Aust NZ J Psychiatry. 2009;43:581-2.
86. Hagerich KL, McNeil MA. Serotonin syndrome: too much of a good thing. J Gen Intern Med. 2009;24:352.
87. Haggerty DA, Curtis J. Serotonin Syndrome Induced Solely by Carisoprodol Overdose. Clin Toxicol. 2010;48:257.
88. Hanekamp BB, Zijlstra JG, Tulleken JE, Ligtenberg JJ, van der Werf TS, Hofstra LS. Serotonin syndrome and rhabdomyolysis in venlafaxine poisoning: a case report. Netherlands J Med. 2005;63:316-8.
89. Hanna ER, Clark JA. Serotonin syndrome after cardiopulmonary bypass: a case demonstrating the interaction between methylene blue and selective serotonin reuptake inhibitors. A A Case Rep. 2014;2:113-4.
90. Hayllar J, Finn J. Serotonin toxicity? The relevance of buprenorphine/naloxone in a curious case of confusion and clonus. Drug Alcohol Rev. 2012;31:71.
91. Hendrix Y, van Zagten MSG. Serotonin syndrome as a result of concomitant use of paroxetine and sumatriptan. Ned Tijdschr Geneeskd. 2005;149:888-90.
92. Heritier Barras AC, Walder B, Seeck M. Serotonin syndrome following Methylene Blue infusion: a rare complication of antidepressant therapy. J Neurol Neurosurg Psychiatry. 2010;81:1412-3.
93. Hernandez-Lorente E, Broto PL, Brumos LG, Simeon Aznar CP. [Serotonin syndrome associated with linezolid]. Med Clin (Barc)2009;132:157-8.
94. Himmighoffen H, Seifritz E, Boeker H. Serotonin Syndrome after Discontinuation of Olanzapine in a Combined Treatment with Duloxetine - Case Report. Pharmacopsychiatry. 2011;44:75-7.
95. Song HK Serotonin Syndrome with Perioperative Oxycodone and Pregabalin. Pain Physician. 2013;16:E632-E3.
96. Houlihan DJ. Serotonin syndrome resulting from coadministration of tramadol, venlafaxine, and mirtazapine. Ann Pharmacother. 2004;38:411-3.
97. Hruby R. Serotonin syndrome - A case report. Int J Neuropsychopharmacol. 2006;9:S217-S8.
98. Hunter B, Kleinert MM, Osatnik J, Soria E. Serotonergic syndrome and abnormal ocular movements: worsening of rigidity by remifentanil? Anesth Analg. 2006;102:1589.
99. Huska MT, Catalano G, Catalano MC. Serotonin syndrome associated with the use of escitalopram. CNS Spectr. 2007;12:270-4.
100. Igneri L, Shaw C, Solomon B. Life-threatening serotonin syndrome after administration of metaxalone in a patient on citalopram. Critical Care Med. 2013;41: A28(12).
101. Inoue T, Watanabe Y, Nodaira Y, Arai J, Sato T, Kikuta T et al. [Peritoneal dialysis patient affected with serotonin syndrome]. Nihon Naika Gakkai zasshi;2008;97:3049-51.
102. Iqbal F, Tsevat J. Serotonin syndrome resulting from concomitant therapy with sertraline and linezolid. J Gen Intern Med. 2009;24:351-2.
103. Isenberg D, Wong SC, Curtis JA. Serotonin syndrome triggered by a single dose of suboxone. Am J Emerg Med. 2008;26:840 e3-5.
104. Ishii M, Tatsuzawa Y, Yoshino A, Nomura S. Serotonin syndrome induced by augmentation of SSRI with methylphenidate. Psychiatry Clin Neurosci. 2008;62:246.
105. Izdes S, Altintas ND, Soykut C. Serotonin syndrome caused by administration of methylene blue to a patient receiving selective serotonin reuptake inhibitors. A A Case Rep. 2014;2:111-2.
106. Jagestedt M, von Bahr C. [Combination of serotonergic agents resulted in severe adverse effects]. Lakartidningen. 2004;101:1618-9.
107. Jang SH, Kwon YM, Chang MC. Serotonin Syndrome in Stroke Patients. J Rehabil Med. 2015;47:282-5 [identified 2014 via e-pub ahead of print ].
108. Jimenez-Genchi A. Immediate switching from moclobemide to duloxetine may induce serotonin syndrome. J Clin psychiatry. 2006;67:1821-2.
109. John AP, Koloth R. Severe serotonin toxicity and manic switch induced by combined use of tramadol and paroxetine. Australian NZ J Psychiatry. 2007;41:192-3.
110. John S, Donnelly M, Uchino K. Catastrophic reversible cerebral vasoconstriction syndrome associated with serotonin syndrome. Headache. 2013;53:1482-7.
111. Joksovic P, Mellos N, van Wattum PJ, Chiles C. "Bath Salts"-Induced Psychosis and Serotonin Toxicity. J Clin Psychiatry. 2012;73:1125.
112. Kaci J, Lowenthal DT, Lagasse S. Clinical physiology and pharmacology conference: rhabdomyolysis and serotonin syndrome in an elderly patient. Int Urol Nephrol. 2007;39:985-7.
113. Kan R, Endou M, Unno Y. [A case of serotonin syndrome following minimum doses of sertraline]. Seishin Shinkeigaku Zasshi. 2009;111:1041-6.
114. Karunatilake H, Buckley NA. Serotonin syndrome induced by fluvoxamine and oxycodone. Ann Pharmacother. 2006;40:155-7.
115. Keegan MT, Brown DR, Rabinstein AA. Serotonin syndrome from the interaction of cyclobenzaprine with other serotoninergic drugs. Anesth Analg.

2006;103:1466-8.

1. Khavandi A, Whitaker J, Gonna H. Serotonin toxicity precipitated by concomitant use of citalopram and methylene blue. Med J Aust. 2008;189:534-5.
2. Kinoshita H, Ohkubo T, Yasuda M, Yakushiji F. Serotonin syndrome induced by dextromethorphan (Medicon) administrated at the conventional dose. Geriatri & Gerontol Int. 2011;11:121-2.
3. Kinzie E, Meltzer-Brody S. Possible serotonin syndrome with citalopram following cross-titration of clozapine to ziprasidone. Gen Hosp Psychiatry. 2005;27:223-4.
4. Kirschner R, Donovan JW. Serotonin syndrome precipitated by fentanyl during procedural sedation. J Emerg Med. 2010;38:477-80.
5. Kirschner RI, Donovan JW. Severe serotonin toxicity treated with intravenous propofol. Clin Toxicol. 2006;44:732-3.
6. Kitson R, Carr B. Tramadol and severe serotonin syndrome. Anaesthesia. 2005;60:934-5.
7. Klys M, Kowalski P, Rojek S, Gross A. Death of a female cocaine user due to the serotonin syndrome following moclobemide-venlafaxine overdose. Forensic Sci Int. 2009;184:e16-20.
8. Klysner R, Bjerg Bendsen B, Hansen MS. Transient serotonin toxicity evoked by combination of electroconvulsive therapy and fluoxetine. Case Rep Psych. 2014;2014:162502.
9. Kohen I, Gordon ML, Manu P. Serotonin syndrome in elderly patients treated for psychotic depression with atypical antipsychotics and antidepressants: two case reports. CNS Spectr. 2007;12:596-8.
10. Kovacic S, Vukovic S, Kocijan-Lovko S, Seferovic M. Combination of fluvoxamine and analgesics can cause serotonin syndrome. Eur J Psychiatry. 2009;23:47-51.
11. Kulkarni RR, Kulkarni PR. Linezolid-induced near-fatal serotonin syndrome during escitalopram therapy: case report and review of literature. Indian J Psychol Med. 2013;35:413-6.
12. Kumar BN, Shah R, Grover S. Serotonin syndrome while switching antidepressants. Indian J Psychiatry. 2011;53:372.
13. 135. Kushwaha S, Panda AK, Malhotra HS, Kaur M. Serotonin syndrome following levodopa treatment in diffuse Lewy body disease. BMJ Case Rep. 2014; doi:10.1136/bcr-2013-201375.
14. Lam PK, Leung KS, Wong TW, Lee HH, Tang MH, Mak TW. Serotonin syndrome following overdose of a non-prescription slimming product containing sibutramine: a case report. Hum Exp Toxicol. 2012;31:414-7.
15. Lamberg JJ, Gordin VN. Serotonin syndrome in a patient with chronic pain polypharmacy. Pain Med. 2014;15:1429-31.
16. Lang PO, Hasso Y, Hilleret H, Vogt-Ferrier N. [Serotonin syndrome as a result of escitalopram and cyclosporin combination in an 84-year-old woman]. Revue Medecine Interne 2008;29:583-6.
17. Larson KJ, Wittwer ED, Nicholson WT, Weingarten TN, Price DL, Sprung J. Myoclonus in patient on fluoxetine after receiving fentanyl and low-dose methylene blue during sentinel lymph node biopsy. J Clin Anesth. 2015;27:247-51.
18. Lattanzi L, Danesi R, Lastella M, Mungai F, Di Paolo A, Tuccori M et al. Serotonin syndrome and the T102-->C polymorphism of the 5-HT2A receptor: a case report. Bipolar Disord. 2008;10:655-6.
19. Lawyer TI, Jensen J, Welton RS. Serotonin Syndrome in the Deployed Setting. Milit Med. 2010;175:950-2.
20. Lee J, Franz L, Goforth HW. Serotonin syndrome in a chronic-pain patient receiving concurrent methadone, ciprofloxacin, and venlafaxine. Psychosomatics. 2009;50:638-9.
21. Levin TT, Cortes-Ladino A, Weiss M, Palomba ML. Life-threatening serotonin toxicity due to a citalopram-fluconazole drug interaction: case reports and discussion. Gen Hospital Psychiatry. 2008;30:372-7.
22. Levine M, Truitt CA, O'Connor AD. Cardiotoxicity and serotonin syndrome complicating a milnacipran overdose. J Med Toxicol. 2011;7:312-6.
23. Liau CH, Shen WW, Su KP. Venlafaxine-associated serotonin syndrome and manic episode in a geriatric depressive patient. Psychiatry Clin Neurosci. 2006;60:121-2.
24. Liberek C, Aubry J-M, Baud P. Manic switch and serotonin syndrome with venlafaxine-lithium-valproate association. Therapie. 2006;61:531-3.
25. Lin PY, Hong CJ, Tsai SJ. Serotonin syndrome caused by ziprasidone alone. Psychiatry Clin Neurosci. 2010;64:338-9.
26. Llinares-Tello F, Escriva-Moscardo S, Martinez-Pastor F, Martinez-Mascaraque P. Possible serotoninergic syndrome associated with coadministration of paroxetine and tramadol. Med Clin (Barc) 2007;128:438.
27. Lopez AM, Kornegay J, Hendrickson RG. Serotonin toxicity associated with Garcinia cambogia over-the-counter supplement. J Med Toxicol. 2014;10:399-401.
28. Lorenz RA, Vandenberg AM, Canepa EA. Serotonergic antidepressants and linezolid: A retrospective chart review and presentation of cases. Int J Psychiatr Med. 2008;38:81-90.
29. 151. Lorenzini KI, Calmy A, Ambrosioni J, Assouline B, Daali Y, Fathi M et al. Serotonin syndrome following drug-drug interactions and CYP2D6 and CYP2C19 genetic polymorphisms in an HIV-infected patient. AIDS. 2012;26:2417-8.
30. Ma J, Zhu P, Tu G, Li X. Serotonin syndrome under combination of linezolid and low-dose citalopram with amiodarone. Psychiatry Clin Neurosci. 2013;67:457.
31. Madsen JM, Curtis JA. An Unusual Case of Serotonin Toxicity. Clin Toxicol. 2010;48:254.
32. Mahlberg R, Kunz D, Sasse J, Kirchheiner J. Serotonin syndrome with tramadol and citalopram. Am J Psych 2004;161:1129.
33. Majewska M, Szponar J, Pyra E, Kostek H, Kujawa A. Serotonin syndrome in the course of drug-poisoning--case presentation. Przegl Lek. 2011;68:523-6.
34. Malik HU, Kumar K. Serotonin syndrome with escitolapram and concomitant use of cocaine: a case report. Clin Med Insights Case Reports. 2012;5:81-5.
35. Marlowe K, Schirgel D. Quetiapine and citalopram: aetiological significances in serotonin syndrome. NZ Med J 2006;119:U2058.
36. Mathew S, Linhartova L, Raghuraman G. Hyperpyrexia and prolonged postoperative disorientation following methylene blue infusion during parathyroidectomy. Anaesthesia. 2006;61:580-3.
37. McClean M, Walsh JC, Condon F. Serotonin syndrome in an orthopaedic patient secondary to linezolid therapy for MRSA infection. Ir J Med Sci. 2011;180:285-6.
38. McDonnell AM, Rybak I, Wadleigh M, Fisher DC. Suspected serotonin syndrome in a patient being treated with methylene blue for ifosfamide encephalopathy. J Onc Pharm Pract. 2012;18:436-9.
39. Miller DG, Lovell EO: Antibiotic-induced serotonin syndrome. J Emerg Med. 2011;40:25-7.
40. Misselbrook GP, Shekhar R. Serotonin syndrome: an unusual cause of acute confusion and fever in the elderly. Acute Med. 2011;10:206-8.
41. Montane E, Barriocanal A, Isern I, Parajon T, Costa J. Multiple drug interactions - induced serotonin syndrome: a case report. J Clin Pharm Ther. 2009;34:485-7.
42. Montanes-Rada F, Bilbao-Garay J, de Lucas-Taracena MT, Ortiz-Ortiz ME. Venlafaxine, serotonin syndrome, and differential diagnoses. J Clin Psychopharmacology. 2005;25:101-2.
43. Monte AA, Chuang R, Bodmer M. Dextromethorphan, chlorphenamine and serotonin toxicity: case report and systematic literature review. Br J Clin Pharmacol. 2010;70:794-8.
44. Monte AA, Waksman JC. Chronic olanzapine, serotonin receptors, and subsequent serotonin toxicity. J Clin Psychopharmacol. 2010;30:628-9.
45. Monterrubio Villar J, Cordoba Lopez A. Serotoninergic syndrome after the administration of clomipramine tablet in a critical patient. Med Intensiva 2007;31:343-4.
46. Morales N, Vermette H. Serotonin syndrome associated with linezolid treatment after discontinuation of fluoxetine. Psychosomatics. 2005;46:274-5.
47. Moseson E, Nichols D. The Clinical Roller Coaster: Severe Serotonin Syndrome. Critical Care Med. 2013; doi:10.1097/01.ccm.0000440576.51720.18.
48. Mugele J, Nanagas KA, Tormoehlen LM. Serotonin syndrome associated with MDPV use: a case report. Ann Emerg Med 2012;60:100-2.
49. Munhoz RP. Serotonin syndrome induced by a combination of bupropion and SSRIs. Clin Neuropharmacol. 2004;27:219-22.
50. Muzyk AJ, Jakel RJ, Preud'homme X. Serotonin syndrome after a massive overdose of controlled-release paroxetine. Psychosomatics. 2010;51:437-42.
51. Nadkarni GN, Hoskote SS, Piotrkowski J, Annapureddy N. Serotonin Syndrome, Disseminated Intravascular Coagulation, and Hepatitis After a Single Ingestion of MDMA in an Asian Woman. Am J Ther. 2012; 21:e117-9.
52. Naka T, Jones D, Baldwin I, Fealy N, Bates S, Goehl H et al. Myoglobin clearance by super high-flux hemofiltration in a case of severe rhabdomyolysis: a case report. Crit Care. 2005;9:R90-5.
53. Nakayama H, Umeda S, Nibuya M, Terao T, Nisijima K, Nomura S. Two cases of mild serotonin toxicity via 5-hydroxytryptamine 1A receptor stimulation. Neuropsychiatr Dis Treat. 2014;10:283-7.
54. Navarro A, Perry C, Bobo WV. A case of serotonin syndrome precipitated by abuse of the anticough remedy dextromethorphan in a bipolar patient treated with fluoxetine and lithium. Gen Hosp Psychiatry. 2006;28:78-80.
55. Nayyar N. Serotonin syndrome associated with sertraline, trazodone and tramadol abuse. Indian J Psychiatry. 2009;51:68.
56. Nefcy A, Wilson J, Smith MP, Maso K, Bora K. Which reality is this? A novel PCP analog combined with 2C-NBOMe causes a dissociative serotonin syndrome. Clin Toxicol. 2013;51:665.
57. Newey CR, Khawam E, Coffman K. Two Cases of Serotonin Syndrome with Venlafaxine and Calcineurin Inhibitors. Psychosomatics. 2011;52:286-90.
58. Ng BK, Cameron AJ, Liang R, Rahman H. [Serotonin syndrome following methylene blue infusion during parathyroidectomy: a case report and literature review]. Can J Anaesth. 2008;55:36-41.
59. Okamoto N, Sakamoto K, Nagafusa Y, Ichikawa M, Nakai T, Higuchi T. Electroconvulsive therapy as a potentially effective treatment for severe serotonin syndrome: two case reports. J Clin Psychopharmacol. 2010;30:350-2.
60. Okamoto N, Sakamoto K, Yamada M. Transient serotonin syndrome by concurrent use of electroconvulsive therapy and selective serotonin reuptake inhibitor: a case report and review of the literature. Case Rep Psychiatry. 2012;2012:215214.
61. Ozdemir S, Yalug I, Aker AT. Serotonin syndrome associated with sertraline monotherapy at therapeutic doses. Progress in neuro-psychopharmacology & biological psychiatry. 2008;32:897-8.
62. Ozkardesler S, Gurpinar T, Akan M, Koca U, Sarikaya H, Olmez T et al. A possible perianesthetic serotonin syndrome related to intrathecal fentanyl. J Clin Anesth. 2008;20:143-5.
63. Palekar N, Eisman J. Serotonin Syndrome With Ziprasidone and Sertraline. J Neuropsychiatry Clin Neurosci. 2013;25:E1-E.
64. Park YM, Jung YK. Manic switch and serotonin syndrome induced by augmentation of paroxetine with methylphenidate in a patient with major depression. Prog Neuropsychopharmacology Biol Psychiatry. 2010;34:719-20.
65. Paruchuri P, Godkar D, Anandacoomarswamy D, Sheth K, Niranjan S. Rare case of serotonin syndrome with therapeutic doses of paroxetine. Am J Ther. 2006;13:550-2.
66. Passmore MJ, Devarajan S, Ghatavi K, Gardner DM, Kutcher SP. Serotonin syndrome with prolonged dysphagia. Can J Psychiatry. 2004;49:79-80.
67. Peacock LE, Wright F. Serotonin syndrome secondary to tramadol and citalopram. Age Ageing. 2011;40:528.
68. Pearce S, Ahned N, Varas GM. A case study of delayed serotonin syndrome: lessons learned. Consult Pharm. 2009;24:64-8.
69. Pilgrim JL, Gerostamoulos D, Woodford N, Drummer OH. Serotonin toxicity involving MDMA (ecstasy) and moclobemide. Forensic Sci Int. 2012;215:184-8.
70. Poeschla BD, Bartle P, Hansen KP. Serotonin syndrome associated with polypharmacy in the elderly. Gen Hospital Psychiatry. 2011;33:301 e9-11.
71. Pollack G, Pollack A, Delfiner J, Fernandez J. Parathyroid surgery and methylene blue: a review with guidelines for safe intraoperative use. The Laryngoscope. 2009;119:1941-6.
72. Prakash S, Belani P, Trivedi A. Headache as a presenting feature in patients with serotonin syndrome: a case series. Cephalalgia 2014;34:148-53.
73. Prakash S, Gosai F, Brahmbhatt J, Shah C. Serotonin syndrome in patients with peripheral neuropathy: a diagnostic challenge. Gen Hospital Psychiatry. 2014;36:450 e9-11.
74. Prator BC. Serotonin syndrome. J Neurosci Nurs: 2006;38:102-5.
75. Primeau M, Pomeraniec F, Wallace DM. Serotonin Toxicity in Aripiprazole Augmentation. J Neuropsychiatry Clin Neurosci. 2012;24:E36-E7.
76. Proudfoot M, Gormley J. Serotonin syndrome: pills, thrills and shoulder aches. BMJ Case Rep. 2013; doi:10.1136/bcr-2012-008314.
77. Rahim MT, Jasti H. Shivers and tremors: A case of serotonin syndrome. J Gen Intern Med. 2006;21:267-8.
78. Rajapakse S, Abeynaike L, Wickramarathne T. Venlafaxine-associated serotonin syndrome causing severe rhabdomyolysis and acute renal failure in a patient with idiopathic Parkinson disease. J Clin Psychopharmacol. 2010;30:620-2.
79. Rang ST, Field J, Irving C. Serotonin toxicity caused by an interaction between fentanyl and paroxetine. Can J Anaesth 2008;55:521-5.
80. Rastogi R, Swarm RA, Patel TA. Case scenario: opioid association with serotonin syndrome: implications to the practitioners. Anesthesiology. 2011;115:1291-8.
81. Reich M, Lefebvre-Kuntz D. Serotoninergic antidepressants and opiate analgesics: A sometimes-painful association. A case report. Enceph-Rev Psychiatr Clin Biol Ther. 2010;36:D119-D23.
82. Riley B. Serotonin syndrome in a patient treated with linezolid. Clin Toxicol. 2005;43:632-3.
83. Rim CL, Gitlin MJ. Ziprasidone, monoamine oxidase inhibitors, and the serotonin syndrome. J Clin Psychopharmacol. 2010;30:470-1.
84. Rittmannsberger H, Werl R. Does aripiprazole protect from serotonin syndrome? Psychiatr Danub. 2012;24:100-1.
85. Rowley M, Riutort K, Shapiro D, Casler J, Festic E, Freeman WD. Methylene blue-associated serotonin syndrome: a 'green' encephalopathy after parathyroidectomy. Neurocrit Care. 2009;11:88-93.
86. Roy B, Massie FS. The Tipping Point: Methadone as a Trigger for serotonin Syndrome. J Gen Intern Med. 2011;26:S455-S6.
87. Sahiner V, Erden Aki SO. [Serotonin syndrome associated with linezolid use: a case report]. Turk psikiyatri 2009;20:398-402.
88. Samartzis L, Savvari P, Kontogiannis S, Dimopoulos S. Linezolid is associated with serotonin syndrome in a patient receiving amitriptyline, and fentanyl: a case report and review of the literature. Case Rep Psychiatry. 2013; doi:10.1155/2013/617251.
89. Sanyal D, Chakraborty S, Bhattacharyya R. An interesting case of serotonin syndrome precipitated by escitalopram. Indian J Pharmacol. 2010;42:418-9.
90. Sartorius A, Wolf J, Henn FA. Lithium and ECT--concurrent use still demands attention: three case reports. World J Biol Psychiatry. 2005;6(2):121-4.
91. Sato A, Okura Y, Minagawa S, Ohno Y, Fujita S, Kondo D et al. Life-threatening serotonin syndrome in a patient with chronic heart failure and CYP2D6*1/*5. Mayo Clin Proc 2004;79:1444-8.
92. Satoh K, Takano S, Onogi T, Ohtsuki K, Kobayashi T. Serotonin syndrome caused by minimum doses of SSRIS in a patient with spinal cord injury. Fukushima J Med Sci. 2006;52:29-33.
93. Schwartz AR, Pizon AF, Brooks DE. Dextromethorphan-induced serotonin syndrome. Clin Toxicol. 2008;46:771-3.
94. Schwiebert C, Irving C, Gillman PK. Small doses of methylene blue, previously considered safe, can precipitate serotonin toxicity. Anaesthesia. 2009;64:924.
95. Sethi R, Kablinger AS, Kavuru B. Serotonin Syndrome in a Sertraline-Treated Man Taking NyQuil Containing Dextromethorphan for Cold. Prim Care Companion CNS Disord. 2012; doi:10.4088/PCC.12l01388.
96. Shahani L. Tramadol precipitating serotonin syndrome in a patient on antidepressants. J Neuropsychiatry Clin Neurosci. 2012;24:E52.
97. Shahani L. Venlafaxine augmentation with lithium leading to serotonin syndrome. J Neuropsychiatry Clin Neurosci. 2012;24:E47.
98. Shaikh ZS, Krueper S, Malins TJ. Serotonin syndrome: take a closer look at the unwell surgical patient. Ann R Coll Surg Engl. 2011;93:569-72.
99. Shakoor M, Ayub S, Ahad A, Ayub Z. Transient serotonin syndrome caused by concurrent use of tramadol and selective serotonin reuptake inhibitor. Am J Case Rep. 2014;15:562-4.
100. Shanmugam G, Kent B, Alsaiwadi T, Baskett R. Serotonin syndrome following cardiac surgery. Interact Cardiovasc Thorac Surg. 2008;7:656-7.
101. Shioda K, Nisijima K, Nishida S, Kato S. Possible serotonin syndrome arising from an interaction between caffeine and serotonergic antidepressants. Human psychopharmacol. 2004;19:353-4.
102. Shopes E, Gerard W, Baughman J. Methylene blue encephalopathy: a case report and review of published cases. AANA J. 2013;81:215-21.
103. Simpson SE, Greenberg MI. Serotonin syndrome associated with ramelteon overdose. Clin Toxicol. 2007;45:630-1.
104. Slettedal JK, Nilssen DO, Magelssen M, Loberg EM, Maehlen J. Brain pathology in fatal serotonin syndrome: presentation of two cases. Neuropathology 2011;31:265-70.
105. Smith C, Marshall SW, Crouch B, Caravati EM. Serotonin Syndrome Precipitated by Methylene Blue. Clin Toxicol. 2010;48(6):664.
106. Somes J, Donatelli NS. Serotonin syndrome-muscle rigidity and confusion in the older adult. J Emerg N : official publication of the Emergency Department Nurses Association. 2012;38:76-8.
107. Stevenson E, Schembri F, Green DM, Burns JD. Serotonin syndrome associated with clozapine withdrawal. JAMA Neurol. 2013;70:1054-5.
108. Stewart DE. Venlafaxine and sour date nut. Am J Psych. 2004;161:1129-30.
109. Stinnett A, Neill K. Case report - serotonin syndrome resulting from exposure to venlafaxine during acute renal insufficiency. Critical Care Med. 2009;37(12):A511-A.
110. Strouse TB, Kerrihard TN, Forscher CA, Zakowski P. Serotonin syndrome precipitated by linezolid in a medically ill patient on duloxetine. J Clin Psychopharmacology. 2006;26:681-3.
111. Szakaly B, Strauss R. Serotonin syndrome in the oral and maxillofacial surgery office: a review of the literature and report of a case. J Oral Maxillofac Surg. 2008;66:1949-52.
112. Szolics M, Chaudhry M, Ljubisavljevic M, Corr P, Samir HA, van Gorkom KN. Neuroimaging findings in a case of fluoxetine overdose. J Neuroradiol. 2012;39:254-7.
113. Tahir N. Serotonin syndrome as a consequence of drug-resistant infections: an interaction between linezolid and citalopram. J Am Med DirAssoc. 2004;5:111-3.
114. Takeshita J, Litzinger MH. Serotonin syndrome associated with tramadol. Prim Care Companion J Clin Psych. 2009;11:273.
115. Talarico G, Tosto G, Pietracupa S, Piacentini E, Canevelli M, Lenzi GL et al. Serotonin toxicity: a short review of the literature and two case reports involving citalopram. Neurol Sci. 2011;32:507-9.
116. Tanaka T, Takasu A, Yoshino A, Terazumi K, Ide M, Nomura S et al. Diphenhydramine overdose mimicking serotonin syndrome. Psychiatry Clin Neurosci. 2011;65:534.
117. Taylor JJ, Wilson JW, Estes LL. Linezolid and serotonergic drug interactions: a retrospective survey. Clin Infectious diseases : an official publication of the Infectious Diseases Society of America. 2006;43:180-7.
118. Terao T, Hikichi T. Serotonin syndrome in a case of depression with various somatic symptoms: The difficulty in differential diagnosis. Prog Neuro-psychopharmacol Biol Psych. 2007;31:295-6.
119. Tiamfook TO, Biddinger PD, Brown DF, Nadel ES. Myoclonus and tachycardia. J Emerg Med. 2005;28:211-4.
120. Top WM, Gillman PK, de Langen CJ, Kooy A. Fatal methylene blue associated serotonin toxicity. Neth J Med. 2014;72:179-81.
121. Torre LE, Menon R, Power BM. Prolonged serotonin toxicity with proserotonergic drugs in the intensive care unit. Crit Care Resusc. 2009;11:272-5.
122. Tseng W-P, Tsai J-H, Wu M-T, Huang C-T, Liu H-W. Citalopram-induced serotonin syndrome: a case report. Kaohsiung J Med Sci. 2005;21:326-8.
123. Turedi S, Eraydin I, Gunduz A, Kalkan A, Hos U. First time, low dose citalopram use-related serotonin syndrome. Neurotoxicology. 2007;28:1272-4.
124. Vari G, Beckson M. Escitalopram-associated serotonin toxicity. J Clin Psychopharmacol. 2007;27:229-30.
125. Velez LI, Shepherd G, Roth BA, Benitez FL. Serotonin syndrome with elevated paroxetine concentrations. Ann Pharmacother. 2004;38:269-72.
126. Verre M, Bossio F, Mammone A, Piccirillo M, Tancioni F, Tortorella V et al. Serotonin syndrome caused by olanzapine and clomipramine. Minerva Anestesiol. 2008;74:41-5.
127. Vinetti M, Duprez T, Philippe H. Severe postoperative hyperthermic syndrome after addition of tilidine/naloxone to duloxetine therapy. Clin Toxicol. 2013;51:516-7.
128. Walter C, Ball D, Duffy M, Mellor JD. An unusual case of serotonin syndrome with oxycodone and citalopram. Case Rep Oncol Med. 2012; doi:10.1155/2012/261787.
129. Warrick BJ, Wilson J, Hedge M, Freeman S, Leonard K, Aaron C. Lethal serotonin syndrome after methylone and butylone ingestion. J Med Toxicol. 2012;8:65-8.
130. Weibrecht KW, Boyer EW. Fluoxetine and 3,4-Methylenedioxymethamphetamine Induced Serotonin Syndrome Responsive to Propofol Therapy. Clin Toxicol. 2010;48:247.
131. Whipp MJ, Waterfield KE. Serotonin syndrome in the differential diagnosis of spinal cord compression. Palliat Med. 2004;18:69-70.
132. Wiegand TJ. A cathinone of a different color - two cases of bupropion abuse presenting with seizures and serotonin syndrome. Clin Toxicol. 2013;51:349-50.
133. Wilson L, Rooney T, Baugh RF, Millington B. Recognition and management of perioperative serotonin syndrome. Am J Otolaryng. 2012;33:319-21.
134. Wood KL, Krishna CV, Thompson JP. Serotonin syndrome following lamotrigine overdose. Clin Toxicol. 2008;46:367.
135. Wu ML, Deng JF. Serotonin toxicity caused by moclobemide too soon after paroxetine-selegiline. JCMA. 2009;72:446-9.
136. Wu ML, Deng JF. Fatal serotonin toxicity caused by moclobemide and fluoxetine overdose. Chang Gung Med J. 2011;34:644-9.
137. Yacoub HA, Johnson WG, Souayah N. Serotonin syndrome after administration of milnacipran for fibromyalgia. Neurology. 2010;74:699-700.
138. Yates SJ, Ahuja N, Gartside SE, McAllister-Williams RH. Serotonin Syndrome following Introduction of Venlafaxine following Withdrawal of Phenelzine: Implications for Drug Washout Periods. Ther Adv Psychopharmacology. 2011;1:125-7.
139. Yee AH, Wijdicks EFM. A Perfect Storm in the Emergency Department. Neurocrit Care. 2010;12:258-60.
140. Young P, Finn BC, Alvarez F, Verdaguer MF, Bottaro FJ, Bruetman JE. Serotonin syndrome: four report cases and review of the literature. An Med Interna. 2008;25:125-30.
141. Zand L, Hoffman SJ, Nyman MA. 74-year-old woman with new-onset myoclonus. Mayo Clin Proc. 2010; 85:955-8.
142. Zonneveld AM, Hagenaars M, Voermans NC, Gelissen HPMM, Claassen JAHR. Life-threatening serotonin syndrome following a single dose of a serotonin reuptake inhibitor during maintenance therapy with a monoamine oxidase inhibitor. Ned tijdschr Geneeskd. 2006;150:1081-4.
